# COVID-19 and Guillain-Barré syndrome: A single-center prospective case series with a 1-year follow-up

**DOI:** 10.1097/MD.0000000000029704

**Published:** 2022-07-29

**Authors:** Lara Ahmad, Pietro Businaro, Simone Regalbuto, Matteo Gastaldi, Elisabetta Zardini, Marta Panzeri, Elisa Vegezzi, Giuseppe Fiamingo, Elena Colombo, Sabrina Ravaglia

**Affiliations:** a IRCCS Mondino Foundation, Pavia, Italy; b ICS Maugeri, Milano, Italy.

**Keywords:** COVID-, 19, Guillain-, Barrè syndrome

## Abstract

Single reports of Guillain-Barré syndrome (GBS) have been reported worldwide during the severe acute respiratory syndrome coronavirus 2 (SARS-CoV-2) pandemic. While case reports are likely to be biased toward uncommon clinical presentations, systematic assessment of prospective series can highlight the true clinical features and spectrum. In this prospective, observational study, we included all consecutive patients who developed GBS. In patients with SARS-CoV-2 infection as antecedent, the time-gap between the infection and GBS onset had to be ≤30 days. The referral was a neurological University Research Hospital, in the Italian Region more severely involved by the pandemic, and hospitalizing both COVID+ and non-COVID neurological diseases. Clinical, laboratory, cerebrospinal fluid, and electromyographic features of GBS diagnosed between March 2020 and March 2021 were compared to a retrospective series of GBS diagnosed between February 2019 and February 2020 (control population). Nasopharyngeal swab was still positive at GBS onset in 50% of patients. Mild-to-moderate COVID-related pneumonia, as assessed by X-ray (6 patients) or X-ray plus computerized tomography (2 patients) co-occurred in 6 of 10 patients. GBS diagnosed during the pandemic period, including 10 COVID-GBS and 10 non–COVID-GBS, had higher disability on admission (*P* = .032) compared to the GBS diagnosed between February 2019 and 2020, possibly related to later hospital referral in the pandemic context. Compared to non–COVID-GBS (n = 10) prospectively diagnosed in the same period (March 2020–2021), post–COVID-GBS (n = 10) had a higher disability score on admission (*P* = .028), lower sum Medical Research Council score (*P* = .022) and lymphopenia (*P* = .025), while there were no differences in GBS subtype/variant, severity of peripheral involvement, prognosis and response to treatment. Cerebrospinal fluid search for SARS-CoV-2 RNA and antiganglioside antibodies were negative in all COVID+ patients. Temporal clustering of cases, coinciding with the waves of the pandemic, and concomitant reduction of the incidence of COVID-negative GBSs may indicate a role for SARS-CoV-2 infection in the development of GBS, although the association may simply be related to a bystander effect of systemic inflammation; lack of prevalence of specific GBS subtypes in post–COVID-GBS also support this view. GBS features and prognosis are not substantially different compared to non–COVID-GBS.

## 1. Introduction

Our group reported the first case series of post-COVID Guillain-Barrè (GBS-C+), collected between March and April 2020 during the first wave of the pandemic in Italy^[1]^; GBS-C+ was later defined as “the first documented COVID-19–triggered autoimmune neurologic disease.”^[2]^ The main information contained in our report^[1]^ included the following: the neurological syndrome may occur both as a post-infectious and a para-infectious event; the peripheral involvement is not specific, but the full spectrum of GBS variants may occur; the development of the peripheral complication may be independent of the severity of respiratory involvement related to severe acute respiratory syndrome coronavirus 2 (SARS-CoV-2) pneumonia, if any; the respiratory failure due to the neuromuscular disease may unmask an otherwise unexplained worsening of respiratory function in a patient hospitalized for pneumonia, in whom, moreover, motor symptoms may go under-recognized. To date, a huge amount of case reports and reviews from several countries have largely replicated and somewhat confirmed these initial findings. Beyond case reports and reviews, literature data from small case series (>10 subjects) are now available.^[3–6]^ In this view, compared to single case reports, systematic prospective case series are more likely to reflect the true disease clinical picture.

### 1.1. Unsolved issues

Unsolved issues remain, such as the long-term prognosis of these patients, most reports showing a very short follow-up, due to the urgency to publish about a relatively still “new” COVID-19 complication; whether there is a prevalence of peculiar GBS subtypes: indeed, the most recent case reports are likely to be biased toward uncommon presentations, due to the tendency to present and publish new and atypical findings; another unsolved issue is—despite the several reports—the very existence of an association between GBS and COVID-19: Keddie et al^[5]^ conclude that this association is, in fact, controversial, based on the prevalence of GBS in the United Kingdom in 2020 compared to the previous 3 years.

### 1.2. Aim

We are participating to a systematic case series collection from several centers in Northern Italy, as an extension of a previous work pertaining the first 3 months of the pandemic.^[3]^ We here report a series of GBS diagnosed over the first pandemic year in a single neurological center, in one of the cities of Northern Italy more severely and earlier involved by the pandemic, and compare them to a retrospective series of GBS diagnosed over the previous year, as well as to non–COVID-GBS (GBS-C−) diagnosed during the pandemic.

We thus aimed at exploring whether the pandemic context modified the handling, management, and treatment strategies of GBS; exploring the clinical and instrumental features of GBS-C+ compared to GBS-C−.

## 2. Patients and Methods

### 2.1. Study design and patients

Our referral center is a neurological University Research Hospital, in the Italian Region more severely involved by the pandemic, and hospitalizing both COVID+ and non–COVID adult (≥18 years of age) neurological diseases. All consecutive patients with GBS diagnosed between March 2020 and March 2021 were recruited prospectively. Diagnosis of antecedent SARS-CoV-2 infection relied on anamnestic (within the previous 30 days) or still positive SARS-CoV-2 RNA in nasopharyngeal swab (NPS), and a highly suggestive clinical antecedent such as fever, pneumonia (as assessed by consistent symptoms/signs and abnormalities in chest computerized tomography or X-ray), anosmia-ageusia, cough. We annotated the time from systemic infection to neurological onset, the features of the infectious antecedent, the follow-up duration.

We collected retrospectively all GBS diagnosed between February 2019 and February 2020 and used them as controls. Diagnosis of GBS was based on Brighton criteria^[7]^ and we included only patients with Brighton 1 and 2.

We annotated cerebrospinal fluid (CSF) findings (albumin, cells, CSF/serum albumin ratio); imaging data, if available (brain or/and spinal cord magnetic resonance imaging [MRI]); neurological examination: consciousness level, cranial nerve involvement, anosmia/ageusia, presence of sensory impairment, occurrence of dysautonomia (unstable blood pressure, arrhythmia, pupillary dysfunction, diaphoresis or anhydrosis, bladder dysfunction); degree of motor weakness, by the Medical Research Council sum scale (60-0, bilateral assessment of 6 muscle groups at lower and upper limbs, the score ranges 0–60 with 0 as the worst score); disability, by the GBS Disability Score or Hughes’s scale.^[8]^ Laboratory examination included creatine kinase, Lactate dehydrogenase, ferritin, D-dimer, lymphocytes, erythrocyte sedimentation rate, C-reactive protein (CRP), antiganglioside antibody serology. Treatment of both GBS and SARS-CoV-2 infection were annotated.

We excluded critical illness neuro-myopathies and entrapment neuropathies possibly related to prolonged hospitalizations and bed confinement.

Time to the last follow-up assessment was annotated for each patient and follow-up consisted on neurological evaluation with measurement of Medical Research Council sum score and functional disability, and neurophysiological re-assessment.

The study was approved by the Ethical Committee of IRCCS C. Mondino (Comitato Etico Satellite, protocol number P_20200053312, on June 15, 2020).

### 2.2. Statistical analysis

Continuous variables are expressed as mean ± standard deviation, or median values and interquartile range depending on the distribution of the variable. Categorical variables are shown as frequencies and percentages.

Statistical analyses were performed with non-parametrical tests (Mann–Whitney *U* test, χ^2^ test or Fisher exact test) due to the small sample size. The statistical threshold was set at 0.05.

The analysis was performed using V.24.0 of the IBM SPSS software (IBM Corp., Armonk, NY).

## 3. Results

### 3.1. The 2019 and 2020 context

In the period March 2020–2021, we observed 10 patients with GBS-C+ and 10 with GBS-C−. COVID diagnosis relied on positive NPS in 8 of 10 patients; of them, 4 of 10 had had recent (<30 days, by selection criteria) positivity associated with consistent clinical manifestations; 4 of 10 had still positive NPS at the onset of the neurological syndrome. In 2 patients, search for SARS-CoV-2 RNA was negative on both NPS and bronchoalveolar lavage, in spite of a typical antecedent (fever, anosmia, dysgeusia in one patient, and ageusia-anosmia in the other): the positivity was thus documented by positive SARS-CoV-2 IgG serology. Indeed, in these 2 patients, the clinical antecedent had occurred during the first month of the pandemic (March 2020), when, due to the severe Health System burden, Italian ministerial rules indicated that the NPS had to be performed only in patients requiring hospitalization for severe pneumonia.

During the period February 2019–February 2020, we recruited 14 GBS. Comparison of GBS—subtypes and other features—observed in these 2 periods are depicted in Table 1. Comparison of GBS-C+ and GBS-C− is illustrated in Table 2, while single cases of GBS-C+ are in Table 3. Delay in GBS diagnosis occurred in 2 GBS-C+ patients with severe respiratory involvement and requiring hospitalization for pneumonia (patients 7 and 9). Usually, however, diagnostic delay was not different compared to GBS-C− diagnosed both in 2019 and 2020, likely because in most patients severe respiratory involvement due to pneumonia did not occur, and also because the presentation of the neurological syndrome was often characterized by rapid progression.

**Table 1:** Comparison of demographic features, disability on admission, and laboratory and instrumental examinations performed in GBS hospitalized in February 2019–2020 vs March 2020–2021.

	GBS February 2019–2020 n = 14	GBS March 2020–2021 n = 20	Value*,†	*P*
Age (mean ± SD)	58 ± 16.7	64.5 ± 10.72	−2,99†	.17
M:F	11:3	11:9	2004*	.147
MRC score	45.5 ± 13.6	41.7 ± 15.27	0.043†	.462
Hughes score on admission				
1	2	1	5.714	**0.032**
2	8	5		
3	1	4		
4	3	10		
GBS subtypes				
AIDP	7/14	7/10 GBS+, 6/10 GBS-C−		
AMAN/AMSAN	4/14	2/10 GBS+, 3/10 GBS-C−		
Other	3/14 (Miller-Fisher: 2, pharingo-cervico-brachial: 1)	1/10 GBS+ (Miller-Fisher), 1/10 GBS-C− (Miller-Fisher)		
Facial involvement	2/14	7/20	2.045*	.360
Antiganglioside antibodies	Not searched: 6/14	Not searched: 3/20 (all GBS-C+)		
	Negative: 5	Negative: 10/10 GBS-C+, 6/10 GBS-C−		
	Positive: 3 (GM1, GQ1b, MOG)	Positive: 1 GBS-C− (GQ1b)		
Imaging examination				
Brain MRI	4/10 (28%)	5/20 (25%), 1/10 GBS-C+, 4/10 GBS-C−		
Spinal MRI	10/14 (71%)	14/20 (70%), 4/10 GBS-C+, 10/10 GBS-C−		
EMG examination	14	20		
CSF examination	14	20		

**Table 2:** Comparison of GBS-C+ and GBS-C−.

	COVID+ n = 10	COVID− n = 10	Value*,†	*P*
M:F	6:4	4:5	0.202*	.502
Age	67.8 *±* 9.8	61.30 *±* 11.05	1.064†	.289
Sum MRC score on admission	37.8 *±* 17.2	45.6 *±* 12.7	−1.21†	**.022**
Hughes score on admission				
1	0	1	6.800*	**.031**
2	1	4		
3	4	0		
4	5	5		
Facial involvement	5/10	2/10	2.359*	.307
CSF albumin (mg/dL)	52 *±* 37.48	60.1 *±* 37.21	-0.61†	.540
Lymphocytes (count/mm^3^)	1491 *±* 616	2069 *±* 935	−1.2†	**.025**
C-reactive protein (mg/dL)	3.92 *±* 5.8	2.2 *±* 3.4	−1.024*,*	.061
Antiganglioside antibodies positivity	0/10	2/10		
Clinical form of GBS				
AIDP	7	6		
AMAN/AMSAN	2	3		
Miller-Fisher syndrome	1	1		
Plasma exchange after immunoglobulin (IVIg) failure	0	1		
Unfavorable prognosis (6–14 mo): unable to walk unassisted	2/10	2/10		

**Table 3:** Single cases of GBS-C+.

Patient	Age, sex	Time C-N*	C manifestations: still† present at the neurological onset #anamnestic	NPS at neurologic onset	GBS type	CSF albumin (timing, d‡)	Spinal/brain MRI	Treatment	Outcome§ (follow-up, mo)	Other neurological features
1∥	77 F	+7	Dysgeusia† Pneumoniae† Hyperthermia¶	+	AMAN Facial +	-T1: 24 -T15: 101	Root+ Embolic stroke	IVIg 2 cycles, PE avoided due to interstitial pneumoniae and positive NFS	Poor, wheel chair bound, ughes 4 (22 mo)	Confusion DWI+ ischemic lesion
2	58 M	+8	Ageusia¶ Hyperthermia¶		AMSAN	T2: 48	Root+	IVIg	Good, no need for FKT (22 mo)	No
3∥	60 M	+7	Dysgeusia† Hyposmia†	BAL negative	AIDP Facial+	T1: 40	Not done	IVIg PE no per Acinetobacter pneumonia ICU for 2 mo due to neuromuscular respiratory failure, TT, PEG	Good, need for FKT (21 mo)	No
4	55 F	+11	Hyposmia†	-	AIDP	T4: 111	Negative	IVIg	Good, no need for FKT (15 mo)	No
5	65 M	+7	Cough† Dyspnea† (interstitial pneumonia)	+	AIDP	T8: 58	Not done	Spontaneous improvement, no treatment	Good, no need for FKT (15 mo)	Confusion
6	74 M	+2	Cough† Desaturation†	+	MFS	T4: 59	Not done	IVIG	Good, no need for FKT (15 mo)	No
7	78 M	+28	Pneumonia†	+	AIDP+ facial	T30: 27	Not done	IVIg late in course of disease	Poor, need for FKT, but incomplete recovery (able to walk with assistance) (14 mo)	
8	81 F	−5	Pneumonia†	+	AIDP+ facial	T1: 19	Not done	IVIg	Good, need for FKT (13 mo)	
9	57 M	+28	Dysgeusia¶ Pneumonia#	-	AIDP, peroneal	T34: 24	Not done	IVIg	Good, no need for FKT (21 mo)	
10	73 F	+11	Dysgeusia†	+	AIDP+ facial	T11: 79	Not done	IVIg	Good, need for FKT (21 mo)	

As regards, neurological investigations, CSF, and electromyography were performed in all GBS-C+ and GBS-C− patients. In GBS-C+ patients, only 4 of 10 (40%) underwent spinal cord MRI: patients with still positive NPS at the time of the neurological onset did not undergo MRI assessment, likely because this implies bringing the patient out of the COVID unit, thus increasing the infectious risk, and considering that spinal cord MRI is not mandatory for GBS diagnosis.

**Figure 1.**
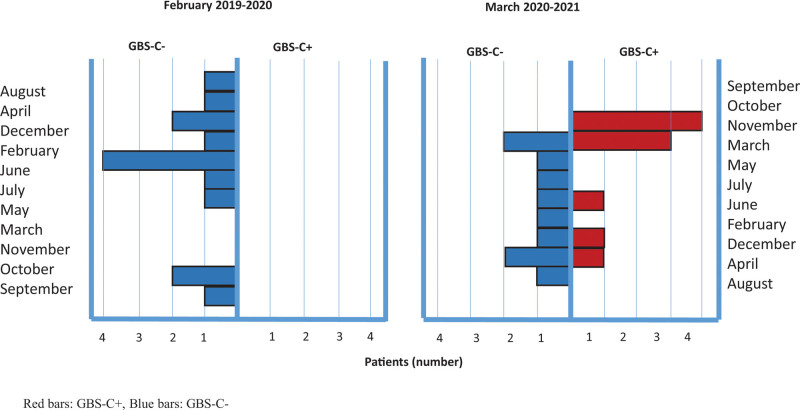
Distribution by onset month of GBS diagnosed between February 2019 and 2020 (retrospective series) and GBS diagnosed between March 2020 and 2021 (prospective series). GBS-C+ (red bars) clusters in March 2020 and November 2020, coinciding with the pandemic waves in our country. Red bars: GBS-C+, blue bars: GBS-C−. GBS = Guillain-Barrè Syndrome; GBS-C+ = post–COVID-19 GBS; GBS-C−: non–COVID-GBS.

As regards the month of onset, in 2019, we observed a clustering of cases in February (5/14), coinciding with the annual spread of influenza, while in 2020, the clustering occurred in March (4/20, ¾ GBS-C+) and in November 2020 (6/20, 4/6 GBS-C+), coinciding with the 2 waves of GBS in our country (Fig. 1). Of the 3 cases that occurred in December 2020, one was GBS-C+, the other 2 cases occurred 3 weeks after influenza vaccination, which was delayed in our country during the last fall season, due to unavailability of the influenza vaccine.

Follow-up duration of patients diagnosed between March 2020 and 2021 ranged 13 to 22 months, mean 17.9 *+* 3.75 months.

### 3.2. Comparison of COVID+ and COVID− GBS

Comparison of GBS-C+ (N = 10), pooled with GBS-C− collected prospectively in the same period (N = 10) and 2019 GBS retrospective series (N = 14) shows, for GBS-C+, higher disability on admission (*P* = .008), higher frequency of facial involvement (*P* = .028), and lower lymphocytes (*P* = .025). Table 2 shows comparison of GBS-C+ and GBS-C− collected prospectively in the period March 2020–March 2021: the same differences were evidenced, but for the same frequency of facial involvement, indicating a possible bias in the detection of a complication with a low clinical impact, such as facial involvement, in the retrospective series.

The infectious antecedent in GBS-C+ occurred a mean of 13 *+* 10.65 (median 9.5, range 2–28) days before the neurological onset. The first referral was neurological for all but 2 patients who had been first hospitalized in non-neurological settings due to pneumonia requiring oxygen therapy (patients 7 and 9).

The polymerase chain reactionPCR search for SARS-CoV-2 in the NPS was still positive at the onset of the neurological syndrome in 4 GBS-C+ patients. CSF search for SARS-CoV-2 RNA was negative in all GBS-C+ patients. Antiganglioside antibodies were negative in all GBS-C+ patients. Facial involvement was prevalent in the GBS-C+ group (5/10 patients, vs 2/10, but the difference did not reach statistical significance); other clinical features (dysautonomia, sensory signs and symptoms, consciousness disturbances) were also similar between the 2 groups.

Among GBS-C+ patients, symptomatic pneumonia with dyspnea or desaturation occurred in 6 patients; only 3 of these patients required oxygen therapy. Thorax X-ray showed mild to moderate involvement (ground-glass opacities and/or reticulation <50%). computerized tomographyCT was performed in 2 patients only, and showed ground-glass opacities <50%. Seven patients received azithromycin, none received chloroquine or antiviral agents, 2 patients received concomitant steroids, and 4 patients received heparin, either for concomitant pneumonia or for the prevention of venous thrombosis.

Two patients developed neuromuscular respiratory failure due to GBS: of them, 1 patient required invasive ventilation and 1 patient required non-invasive ventilation. Concomitant radiological signs of pneumonia were mild in 1 patient (patient 1) and absent in the other (patient 3).

Lymphopenia was more common in GBS-C+ (*P* = .025), and persisted even when the neurologic onset had followed the resolution of the antecedent infection, while CRP was at the limits of significance (*P* = .061).

GBS presentation was the classic acute inflammatory demyelinating polyneuropathy (AIDP) in 7 patients, acute motor axonal neuropathy (AMAN) in 1 patient, acute motor-sensory axonal neuropathy (AMSAN) in 1 patient, and Miller-Fisher Syndrome in 1 patient. The proportion of AIDP and AMAN and rarer variants was not substantially different from the retrospective series (Tables 1 and 2).

Brighton criteria was 1 in all but 3 patients who were actually Brighton 2 due to negative CSF, likely because CSF analysis was too precocious (e.g., patient 1, Table 3, repeated CSF 15 days after T1 showed increased albumin and normal cells, so that this patient was later classified as Brighton 1) or too tardive (patients 7 and 9 who were diagnosed late in the course of disease).

All patients received IVIg, with the exception of one patient (patient 5) who showed spontaneous recovery. For the 2 patients with a null response to IVIg (patients 1 and 3), plasma exchange (PE) was not proposed, due to concomitant SARS-CoV-2–related pneumonia (patient 1) and superinfection by Aspergillus in patient 3. A repeated cycle of IVIg was thus attempted, 14 (patient 1) and 21 days (patient 3) after the end of the first IVIg course, after repeating CSF analysis that still showed persistency of raised albumin, likely indicating a still active inflammatory process.

No deaths occurred. Long-term prognosis was generally good, although recovery could take as long as 6 months, due to the often severe disability, with the exception of 2 patients: patient 1, with severe axonal damage (AMAN), who is still confined to wheelchair 22 months year after the onset, and patient 7, who was likely treated late in the course of disease (in this patient AIDP was suspected when facial involvement and dysphagia occurred, about 30 days form the onset of muscle weakness, that had been initially attributed to bed confinement due to pneumonia). Table 3 shows details of single GBS-C+ patients.

## 4. Discussion

### 4.1. Our context and our selection criteria

Our series of GBS associated with SARS-CoV-2 was collected in a single center that, during the pandemic, hospitalized both COVID-positive and COVID-negative patients, possibly reducing any diagnostic bias related to non-neurological referrals. Our center is a University Neurological Hospital located in Lumbardy, the Italian Region more severely and earlier involved by the pandemic.

Diagnosis of GBS relied on Brighton criteria: all patients had Brighton level = 1, with only 2 patients with Brighton level = 2, in both cases due to normal CSF.

To establish a strict relationship with SARS-CoV-2 infection, we chose a short time interval (<30 days) between the documentation of SARS-CoV-2 positivity and the onset of the neurological syndrome. Indeed, at the onset of the neurological syndrome, 4 patients had still positive NPS, 6 had thorax imaging findings still suggestive of recent/active pneumonia, 8 still had lymphopenia (<1.500/mm^3^), and all had still raised CRP.

### 4.2. Relationship between SARS-CoV-2 antecedent and neurological onset

As regards the timing of the neurological onset, in all our patients, the symptoms of GBS began 2 to 28 days after COVID-19 symptoms: in the 4 patients with still positive NPS at the time of the neurological onset, we cannot say if this means still active infection (so that these forms should be considered para-infectious) or rather whether it may be attributed to slow clearance of non-replicating viral genomes from nasopharyngeal mucosae. Six of 10 GBS-C+ also showed pneumonia on thorax imaging, but it is difficult to establish in which stage GBS symptoms occurred, since COVID-19 respiratory symptoms and chest imaging abnormalities may persist beyond the acute infection phase. Indeed, of 6 patients with still positive thorax imaging, 4 had frank concurrent pneumonia symptoms, though mild. Inflammatory indices such as CRP and erythrocyte sedimentation rate were also often still positive at the neurological onset, suggesting that the association may actually be both post- and para-infectious. In 2 patients, there is clear evidence of a post-infectious onset that, moreover, may also occur when the symptoms of the antecedent infection are very mild: in patients 2 and 3, the nasopharingeal swabNFS and bronchoalveolar lavage were negative at the neurological onset, the antecedent infection did not cause pneumonia, thorax X-ray was negative, and the positivity—suggested by antecedent anosmia and dysgeusia—could be detected only by serology.

### 4.3. Comparison of GBS patients diagnosed before and during the pandemic

Patients hospitalized in March 2020 to March 2021 showed higher disability on admission, possibly due to later hospital referral in the context of the pandemic burden of hospitals. Comparing GBS-C+ in 2020 versus GBS-C−, higher disability seems to occur in the GBS-C+ group: it is possible that early symptoms of peripheral involvement such as pain, sensory disturbances, mild motor dysfunction, may have been overlooked due to the concurrent infection; another reason may be that patients with symptoms of (respiratory) infection, but not as severe as to require hospitalization, were instructed to stay home in quarantine and not to search for medical attention except in case of serious need.

In the pandemic period, patients with GBS underwent the same set of investigations as the control group, with the exception of MRI that was performed only in 4 GBS-C+, likely because MRI is not mandatory for diagnosis and contemplates bringing a patient out of the COVID unit with increased infectious risk. On the contrary, investigations that could be performed in the COVID unit, such as electromyography and CSF, or laboratory investigations, were even repeated more times, including a complete serological assessment: for example, antigangliosides were tested in all GBS-C+ patients, maybe indicating higher scientific interest in these forms.

These were no substantial differences in GBS subtypes diagnosed before and during the pandemic (Tables 1 and 2) with a similar prevalence of the most common AIDP (2019: 50%; 2020: 65%) and AMAN/AMSAN (2019: 21%; 2020: 25%).

### 4.4. Comparison of GBS-C+ and GBS-C−

The features of GBS-C+ were similar to GBS-C− as regards the type of GBS syndrome, electrophysiological abnormalities, CSF features, degree of motor impairment; the only true differences were the already mentioned higher onset disability score and the prevalence of facial involvement in GBS-C+. Although frequent detection of facial involvement is in line with literature data,^[4]^ we cannot exclude that the retrospective nature of the control group may create a bias for missing a sign with mild functional impact such as facial palsy. Indeed, prevalence of facial involvement emerged in comparison with the retrospective group only (*P* = .028) but not comparing GBS-C+ and GBS-C− prospectively collected (*P* = .307).

### 4.5. Prognosis and treatment response

Compared to most literature cases, follow-up in our series is longer, ranging 13 to 22 months (17.9 *+* 3.75), so that we could collect a moderately long-term (>1 year) prognosis in all patients. Despite several case reports and small series, there are only few data on the prognosis of GBS-C+. Reviews claim of more severe prognosis, but when exploring single reports we observe that the follow-up is almost invariably shorter than 3 months. In our series, despite higher disability on admission, the final prognosis did not seem different between GBS-C+ and GBS-C−, and was overall good, with only 2 patients not recovering walking abilities (patients 1 and 7), and in one of the 2, this was possibly due to treatment late in the course of disease (patient 7).

The clinical presentation encompassed severe manifestations with neuromuscular respiratory failure (patients 1 and 3) to milder forms, with 1 patient (patient 5) not even requiring IVIg due to spontaneous recovery, and 2 patients receiving IVIg despite mild clinical dysfunction 1 week and 28 days after the neurological onset (patients 2 and 9).

The response to IVIg also did not show significant differences, being satisfactory in all but 2 patients: in these 2 patients, PE was not attempted, either due to persistent NPS positivity and pneumonia (patient 1), with the fear to depress the immune response against COVID-19, or due to bacterial superinfection (patient 3). Indeed, concomitant infection may discourage PE, and literature data on the use of PE as an alternative to IVIg in GBS-C+ are still scarce.^[4]^ Despite the lack of clinical guidelines or other evidence supporting a double IVIG course, we found that this was the only possible therapeutic approach, considering the still severe motor and respiratory involvement of our 2 patients. Indeed, the presence of active infection (COVID-related pneumonia in patient 1 and Aspergillus pneumonia superinfection on patient 3) contraindicated PE and other immunosuppressive approaches; the relatively long temporal distance (14 and 21 days) from the first cycle lead us to consider the second course as acceptably safe; moreover, repeated CSF analysis still showed persistency of raised albumin, likely indicating a still active inflammatory process and thus still active disease.

### 4.6. Implications about pathogenesis

As regards pathogenesis, we can only speculate that the lack of peculiarities of GBS-C+ as regards clinical features, GBS subtype, prognosis, CSF features, neurophysiology, together with lack of specific antibodies, the occurrence both as a para-infectious or a post-infectious event, all suggest that the trigger may be the inflammatory context itself, rather than a specific trigger epitope peculiar to SARS-CoV-2. In this view, a non-specific inflammation, that is, a post-acute hyper inflammatory illness and a dysregulated host response, could trigger the neurological complication; in all our patients, SARS-CoV-2 RNA was absent in the CSF, thus there is no evidence of direct peripheral damage. A dysregulation of the immune response induced by the virus or by a cytokine storm remains the more plausible hypothesis. The lack of antiganglioside antibodies in GBS-C+ also points against molecular mimicry. Lack of genetic similarities between the peripheral nerve myelin and viral genome also supports this view.^[9]^ Indeed, high levels of interleukinIL-6 and other pro-inflammatory cytokines in SARS-CoV-2 infection suggest an intense immunological reaction, and interleukinIL-6 has also been implicated in the development of GBS.^[10]^

### 4.7. Controversies about the association between SARS-CoV-2 and GBS

Recent studies have raised doubts on the association between GBS and COVID^[5,9]^; one of the arguments is the lack of overall increase in incidence of GBS during the pandemic. We believe that studies on the epidemiology of post-COVID GBS should not be based on the global prevalence of GBS during the pandemic period, for 2 reasons:

1. Although this statement should be replicated in larger series and in different areas, our data seem to show a reduction of GBS-C− during the pandemic period.

2. In the pandemic context, both patients with severe pneumonia (i.e., patients 7 and 9 in our series, in whom diagnostic delay due to severe pneumonia occurred), as well as cases with mild neurological disability, can be under-estimated.

First, the number of GBS-C− observed in March 2020 to 2021 is slightly lower than those diagnosed in the previous year. These data should be replicated in multicenter studies and in larger populations and, possibly, in areas differently affected by COVID-19 also. If confirmed, we can hypothesize that previous statements about the lack of association between GBS and COVID-19,^[5]^ pointing to a lack of increase in worldwide cases of GBS during the pandemic, are related to the concomitant reduction of non-COVID GBS. We believe that lockdown and other protective measures reduced the incidence of oro-fecal (*Campylobacter*C. *Jejuni*) and respiratory infections: this observation indirectly reinforces the likely role for COVID-19 itself in triggering GBS. The imbalance between increase in COVID-GBS versus decrease in non–COVID-GBS would also explain the lack of worldwide raise in GBS incidence during the pandemic (although epidemiological information supporting this statement is unavailable till now), that Lunn et al^[9]^ claim as a strong argument against the association. These authors calculated the incidence of GBS by judging the use of IVIg. However, in such an emergency context (Italy was the first country, after China, to be dramatically involved by the outbreak), we believe that many GBS cases could have been missed, that is, poorly symptomatic cases and cases with spontaneous resolution. First, it is easy to overlook the motor syndrome in a context of a dramatic respiratory and systemic life-threatening condition: concomitant neuromuscular and respiratory dysfunction is not easily ascertained in an intensive care unitICU/assisted ventilation setting or in severely ill patients that are confined to bed. Thus, GBS cases attending the ER could have been missed in more severe COVID patients due to severe pneumonia, as occurred in patients 7 and 9; we also could have missed these cases being a neurological referral. Second, milder GBS cases could have been also missed, due to people avoiding hospital attendance (e.g., the same has occurred for milder strokes), as also shown by higher neurological disability at onset during the pandemic period. Actually, underestimation of milder forms of GBS during the pandemic may concern GBS-C− also, but likely less often than GBS-C+, since GBS-C+ also are in quarantine due to recent or concurrent infectious symptoms, and this further discourages hospital admission. This may account for by the severity of neurological disability at onset in GBS-C+ patients. Thus, calculating the incidence of GBS by the analysis of National Health System IVIg registries^[5]^ in a pandemic context may overlook milder cases, but also severe cases may be missed: physician prescription may become more selective and influenced by the expected (bad) outcome of the primary infection, and however many diagnoses may be missed due to comorbid complications.

### 4.8. Temporal clustering of cases

In our series, GBS-C+ seem to cluster in the months coinciding with the spread of infection in Italy, on March 2020 and November 2020, while GBS-C− were overall slightly decreased in frequency and spread over the 12 months, with a tendency to peak in February 2019, during the influenza spread. Although this temporal clustering may indirectly support a pathogenic link between COVID-19 and GBS, the mechanism remains speculative, and a simple bystander effect of systemic inflammation cannot be excluded.

### 4.9. Unsolved (unsolvable?) issues

The true incidence of COVID-19 cannot be ascertained given that the spectrum of the disease include asymptomatic forms. We cannot judge, indeed, the proportion of COVID-19–positive cases by the official numbers of positive NPSs: the true prevalence of COVID-19 is certainly much higher. Thus, the strength of the association between COVID-19 and GBS cannot be established. On the other hand, we are not sure it is correct to establish a link between a rare, unpredictable complication and a common disease by simply assessing the distribution of the trigger in relation to its complication, and considering that the pathogenesis of GBS itself is far from clear. The relationship between the viral trigger and the immune complication may be complex, and genetic or other environmental factors may take a role. Leonhard et al^[11]^ established there was a causal relationship between Zika virus and GBS, simply because Zika outbreaks increased the incidence of GBS in that Countries. However, the context here is different, since Zika is not a pandemic but its spread is limited to distinct areas that have distinct genetic background, as well as other site-specific environmental factors (co-occurrence with other endemic infections which may contribute as triggers for instance). An increased genetic/environmental susceptibility to molecular mimicry or to a hyper-immune response associated with coronaviruses, and thus an increased risk for auto-immune, post-infectious complications, may explain the different incidence reported in different countries. Indeed, review articles^[12]^ report a cluster of cases of post-COVID GBS in some countries, with 40% coming from Italy (and especially Northern Italy) and Spain. A recent large epidemiological case-control study coming from the Spanish population^[4]^ indeed found results similar to our North-Italian population.

### 4.10. Conclusions

Half of the cases of GBS that we observed in the pandemic year followed COVID-19 infection. Higher onset disability may be related to later hospital presentation, due to the hospitals’ burden, and does not seem to be associated with a worse prognosis. Given the absence of specific disease features and of specific GBS subtypes, the association between COVID-19 and GBS has to be supported by further studies on larger groups and on different populations. GBS is known to follow many viral and bacterial infections, but only a few of them are proven. The association with COVID-19 might not be unexpected, considering that GBS is unanimously considered a post-infectious disease with several new infectious agents, even recently added as possible triggers, including Zika Virus and Hepatitis E.^[13,14]^ Although we cannot definitively support a causal link, we believe that COVID-19 is likely to trigger, in a small proportion of susceptible individuals, GBS, although the mechanism remain speculative. The spectrum includes mild and severe forms, as in classical GBS. Long-term follow-up, lacking in most reported series, shows that prognosis also is not different from classical GBS. Our setting is that of a purely neurological referral, that may have missed forms with severe pneumonia. Indeed, controversial results of previous studies may be related to misdiagnosis of forms with mild neurological disability, as well as forms with severe pneumonia, in the pandemic context. We do not conclude for a definite causative relationship between GBS and SARS-CoV-2: our data need to be confirmed by multi-center studies on larger groups, including populations that may have different genetic or environmental susceptibility to develop GBS.

## Author contributions

SRav, LA, EV conceived the study and analyzed results and wrote the paper; EZ, MG performed laboratory investigations; PB, SReg, EC collected the data and performed follow-up assessment of the patients.

## Footnotes

* Chi-square.

† Mann–Whitney *U.*

* Chi-square.

† Mann–Whitney *U.*

* Time C-N: time between COVID-19 symptoms onset, C, and neurological onset, N, in days.

‡ Timing of CSF examination compared to the onset of the neurological syndrome, expressed in days: T-number, followed by albumin value on CSF.

§ Final outcome was labeled as: good = able to walk unassisted, poor: wheelchair bound or walking with assistance.

∥ Already published in reference [1].
